# Buddhist Philosophy and Mental Health: Lessons for the 21st Century

**DOI:** 10.1192/bjo.2022.113

**Published:** 2022-06-20

**Authors:** Patrick Briggs

**Affiliations:** Merseycare NHS Foundation Trust, Liverpool, United Kingdom

## Abstract

**Aims:**

The aim of this research was to highlight the aspects of Buddhist philosophy which may help to improve mental health. COVID-19 has had a considerable psychological impact on healthcare staff and the general population, emphasizing the importance of treatments and techniques to aid their mental health.

**Methods:**

Mindfulness, Impermanence and Non-self were discussed as core aspects of Buddhist philosophy and how these relate to mental well-being. Reference was made to peer-reviewed studies that show the positive effects of these concepts.

**Results:**

This research highlighted the wealth of evidence that Mindfulness, Impermanence and Non-self has in improving mental well-being. However, there were also risks, including depersonalisation and increased anxiety in certain mindfulness practitioners.

**Conclusion:**

The findings of this research has generated new ways in which we discuss mental well-being and challenges our current understanding of suffering, providing individuals with further tools to assist with their mental health. This study challenges the idea that philosophy and medicine must be discussed separately and seeks to find further common ground between these two disciplines.

